# The effects of psyllium husk on gut microbiota composition and function in chronically constipated women of reproductive age using 16S rRNA gene sequencing analysis

**DOI:** 10.18632/aging.203095

**Published:** 2021-06-03

**Authors:** Chuanli Yang, Shuai Liu, Hongxia Li, Xinshu Bai, Shuhua Shan, Peng Gao, Xiushan Dong

**Affiliations:** 1 Department of General Surgery, Shanxi Bethune Hospital (Shanxi Academy of Medical Sciences), Taiyuan, China; 2 Graduate School, Shanxi Medical University, Taiyuan, China; 3 Shanxi Provincial Cancer Hospital, Taiyuan, China; 4 Key Laboratory of Chemical Biology and Molecular Engineering of National Ministry of Education, Institute of Biotechnology, Shanxi University, Taiyuan, China; 5 BGI Life Science Research Institution, Shenzhen, China

**Keywords:** women of reproductive age, chronic constipation, gut microbiota, 16S rRNA gene sequencing, metabolism

## Abstract

Chronic constipation is a common gastrointestinal disorder that occurs in the elderly and in women. Psyllium husk is widely used to treat this condition. Recent studies have shown that psyllium husk can improve the clinical symptoms of constipation by regulating gut microbiota, but its clinical effects and potential mechanisms in constipated women of reproductive age have not been previously investigated. We compared fecal microbiota after treatment with placebo (n = 29) and psyllium husk (n = 25) using 16S ribosomal ribonucleic acid (rRNA) gene sequencing analysis. Psyllium husk relieved the symptoms of constipated women of reproductive age. Sequencing results showed that the psyllium husk group exhibited a different gut microbiota composition compared to that of the placebo group. Moreover, network analysis indicated more significant correlations and clustering of operational taxonomic units (OTUs) in the psyllium husk group. Kyoto Encyclopedia of Genes and Genomes (KEGG) annotation analysis showed that the relative abundances of metabolism-related KEGG pathways were enriched in the psyllium husk group. In conclusion, these findings suggest that the composition of gut microbiota was altered and that symptoms of constipation were alleviated via psyllium husk intervention. The changes in metabolic function might be related to constipation. Furthermore, these studies are warranted to elucidate the potential metabolic mechanisms contributing to chronic constipation.

## INTRODUCTION

Chronic constipation is one of the most common symptoms worldwide; it can occur by itself or can be secondary to other medical conditions [1]. The main characteristics of chronic constipation are difficult passage of stool, reduced frequency of bowel movements (BMs), and a feeling of incomplete defecation. Most previous studies have shown that the prevalence of constipation is 12–19% in adults living in North America [2, 3], whereas this prevalence is 26% in women and 16% in men over the age of 65 [4, 5]. In addition, among people aged ≥84 years, the prevalence of constipation is 34% in women and 26% in men [6–8]. Especially during late pregnancy, the risk of constipation is higher due to reduced intestinal movement, significant hormonal changes, and delayed bowel emptying [9, 10]. However, most of the current research on constipation has focused on all adults, and has only rarely focused specifically on women of reproductive age (women of childbearing ability between the ages of 15 and 49 years) alone. Therefore, there is an urgent need to explore the impact of constipation in such women.

With the development of next-generation sequencing (NGS) technology, recent studies have indicated dysbiosis of gut microbiota in chronic constipation [11]. Owing to the influences of age, sex, and hormones, the findings on gut microbiota in chronic constipation are paradoxical, and, currently, no consensus exists [11]. Fecal microbiota transplantation (FMT), probiotics, prebiotics, synbiotics, and antibiotics have been used to relieve symptoms of chronic constipation [12].

Psyllium is a widely used treatment for constipation [13, 14]. This soluble dietary fiber, derived from the seeds of *Plantago ovata* or *Psyllium plantago,* is a traditional herb and non-fermented fiber supplement [15]. It is a polymer rich in uronic acids, pentoses, and hexoses that has limited digestibility. Owing to its composition, psyllium husk improves water retention capacity in the small intestine and increases the mobility of colonic content [16, 17]. Therefore, it can relieve the symptoms of constipation and is considered to have potential prebiotic effects [18, 19]. Although the effects of psyllium husk on alleviating symptoms of constipation have been described, the role of gut microbiota in this effect has remained unclear.

In the present study, we investigated the effects of psyllium husk on the gut microbiota of constipated women aged 15-49 years using a randomized, parallel, placebo-controlled trial. Herein, we found that psyllium husk relieved the symptoms of constipation, compared with those form baseline data, including the following : < 3 BMs/week (72%), hard stool (36%), pain with bowel movement (24%), feeling of incomplete defecation (56%), feeling of blockage (40%). Moreover, these changes were accompanied by alterations in the composition and function of gut microbiota.

## RESULTS

### Characteristics

A total of 54 patients from both the psyllium husk group and placebo group completed treatment and were available for four weeks. The two treatment groups were commonly matched by age, body mass index (BMI), straining, <3 bowel movements (BMs) / week, hard stool, pain with BMs, feeling of incomplete defecation, digital maneuvering, and feeling of blockage (Table 1). More detailed clinical information is summarized in Supplementary Table 2. The two groups of baseline data were balanced and comparable. Psyllium husk was well tolerated, and no serious adverse events occurred.

**Table 1:** Characteristics of constipated patients of reproductive age by treatment group at baseline.

**Characteristics**	**placebo**	**psyllium husk**	***P*-Value**
Demographics			
n	29	25	
Age (years, mean ± SD)	34.07 ± 6.245	31.16 ± 6.28	0.447
Female subjects	100%	100%	
Body-mass-index (kg/m2, mean ± SD)	21.90 ± 2.88	22.22 ± 2.18	0.329
Clinical symptoms			
Straining (n, %)	15 (62.5)	9 (37.5)	0.256
< 3 BM/week (n, %)	25 (86.2)	23 (92.0)	0.499
Hard stool (n, %)	17 (58.6)	17 (68.0)	0.477
Pain with bowel movement (n, %)	16 (55.2)	13 (52.0)	0.816
Feeling of incomplete defecation (n, %)	19 (65.5)	18 (72.0)	0.609
Digital maneuver (n, %)	1 (3.4)	1 (4.0)	1.000
Fleeing of blockage (n, %)	19 (65.5)	20 (80.0)	0.236

### Psyllium husk relieves the symptoms of constipated women of reproductive age

As shown in Table 2, there were significant differences between the psyllium husk group and placebo group in terms of straining (2, 8.0% vs. 16, 55.2%, *P* = 0.000), <3 BMs/week (5, 20.0% vs. 25, 86.2%, *P* = 0.000), hard stool (8, 32.0% vs. 18, 62.1%, *P* = 0.027), pain with BMs (7, 28.0% vs. 17, 58.6%, *P* = 0.024), and feeling of incomplete defecation (4, 16.0% vs. 19, 65.5%, *P* = 0.000). Digital maneuvering and feeling of blockage were not statistically different between the two groups. Although feeling of blockage did not differ to a statistically significant degree between the groups, the proportion thereof was lower in the psyllium husk group than in the placebo group. These results indicated that psyllium husk as supplementation relieved the symptoms of constipation, and that these therapeutic effects occurred without serious adverse reactions.

**Table 2:** Characteristics of constipated patients measured after 4 weeks of intervention.

**Clinical symptoms**	**placebo (n, %)**	**psyllium husk (n, %)**	***P*-Value**
Straining	16 (55.2)	2 (8.0)	0.000
< 3 BM /week	25 (86.2)	5 (20.0)	0.000
Hard stool	18 (62.1)	8 (32.0)	0.027
Pain with bowel movement	17 (58.6)	7 (28.0)	0.024
Feeling of incomplete defecation	19 (65.5)	4 (16.0)	0.000
Digital maneuver	1 (3.4)	1 (4.0)	1.000
Fleeing of blockage	17 (58.6)	10 (40.0)	0.172

### Taxonomic profiling of gut microbiota in women of reproductive age receiving placebo and psyllium husk intervention using 16S rRNA gene sequencing

**Figure 1:**
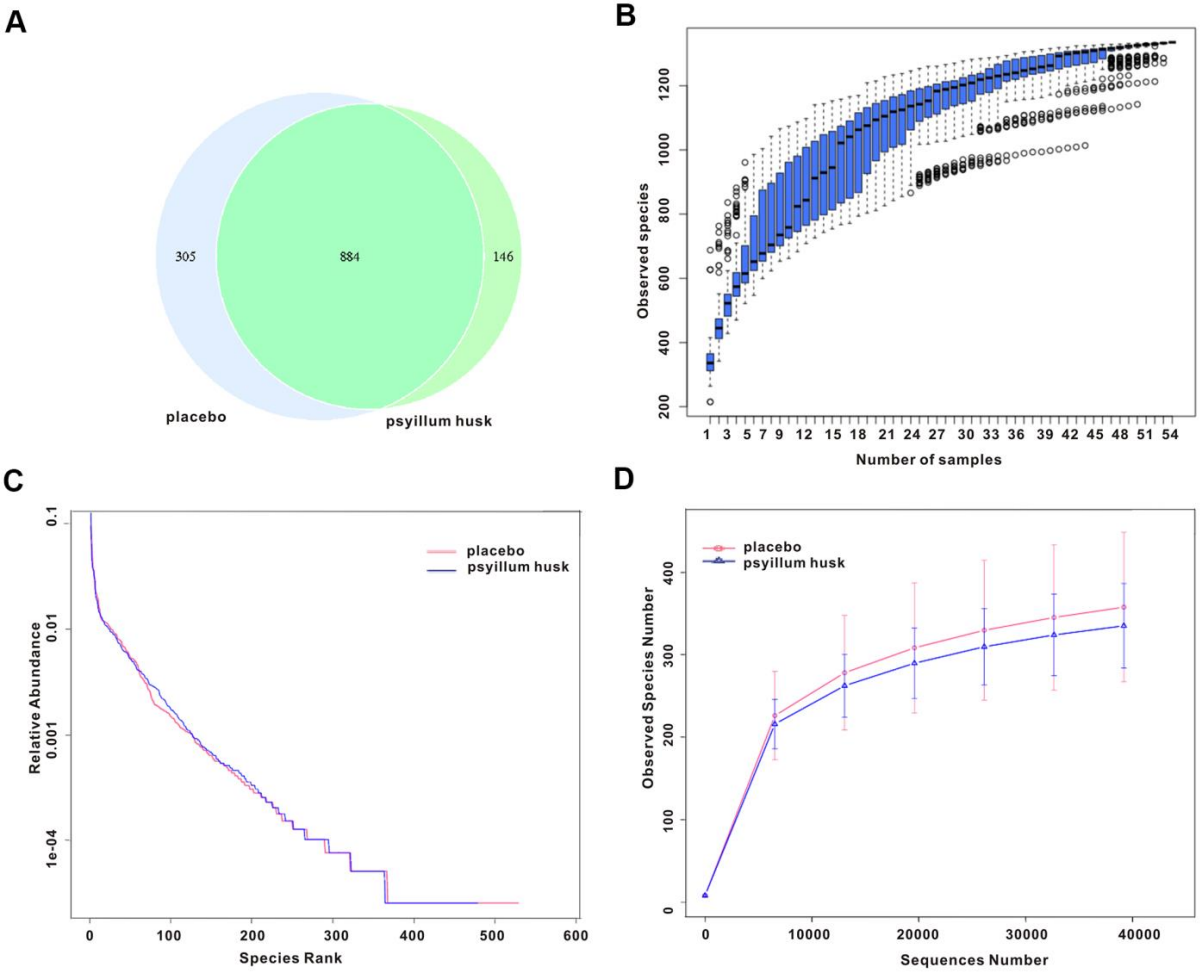
**Species abundance and bacterial diversity in both groups.** (**A**) The Venn diagram indicates overlap of OTUs between the two groups. (**B**) The species accumulation boxplot displays species richness. (**C**, **D**) Rank abundance curve analysis and rarefaction curve analysis were used to show bacterial diversity and species abundance.

Numerous studies have shown that disorders of intestinal flora are among others associated with chronic constipation [11]. To further explore the potential intestinal flora–related mechanisms of psyllium husk in relieving the clinical symptoms of chronic constipation in women of reproductive age, we determined microbiotal compositions via 16S rRNA sequencing. After filtering, quality control, and chimera removal, a total of 4,658,291 effective sequences were obtained from the 54 fecal samples (29 samples from the placebo group, 25 samples from the psyllium husk group). In addition, 1,335 operational taxonomic units (OTUs), which can be used to further analyze species within sample via rapid visualization of species composition and abundance, were matched at 97% identity, including 21 phyla, 37 classes, 74 orders, 126 families, 261 genera, and 230 species of gut microbes that we annotated for subsequent analyses. Venn diagrams showed that the two groups shared 844 common OTUs (Figure 1A). We used a species-accumulation boxplot to evaluate the sufficiency of sample number and species richness. As shown in Figure 1B, when the number of samples reached 54, the number of species observed was nearly parallel, indicating that the sample size of our experiment was sufficient. Rank abundance was used to reflect the richness and uniformity of species in each sample, while the rarefaction curve was used to reflect the rationality of the amount of sequencing data and to indirectly reflect species abundance in each sample. The rank abundance curve of the psyllium husk group was slightly lower than that of the placebo group, but this difference was not statistically significant (Figure 1C). Furthermore, the rarefaction curve had similar trends in both groups (Figure 1D).

**Figure 2:**
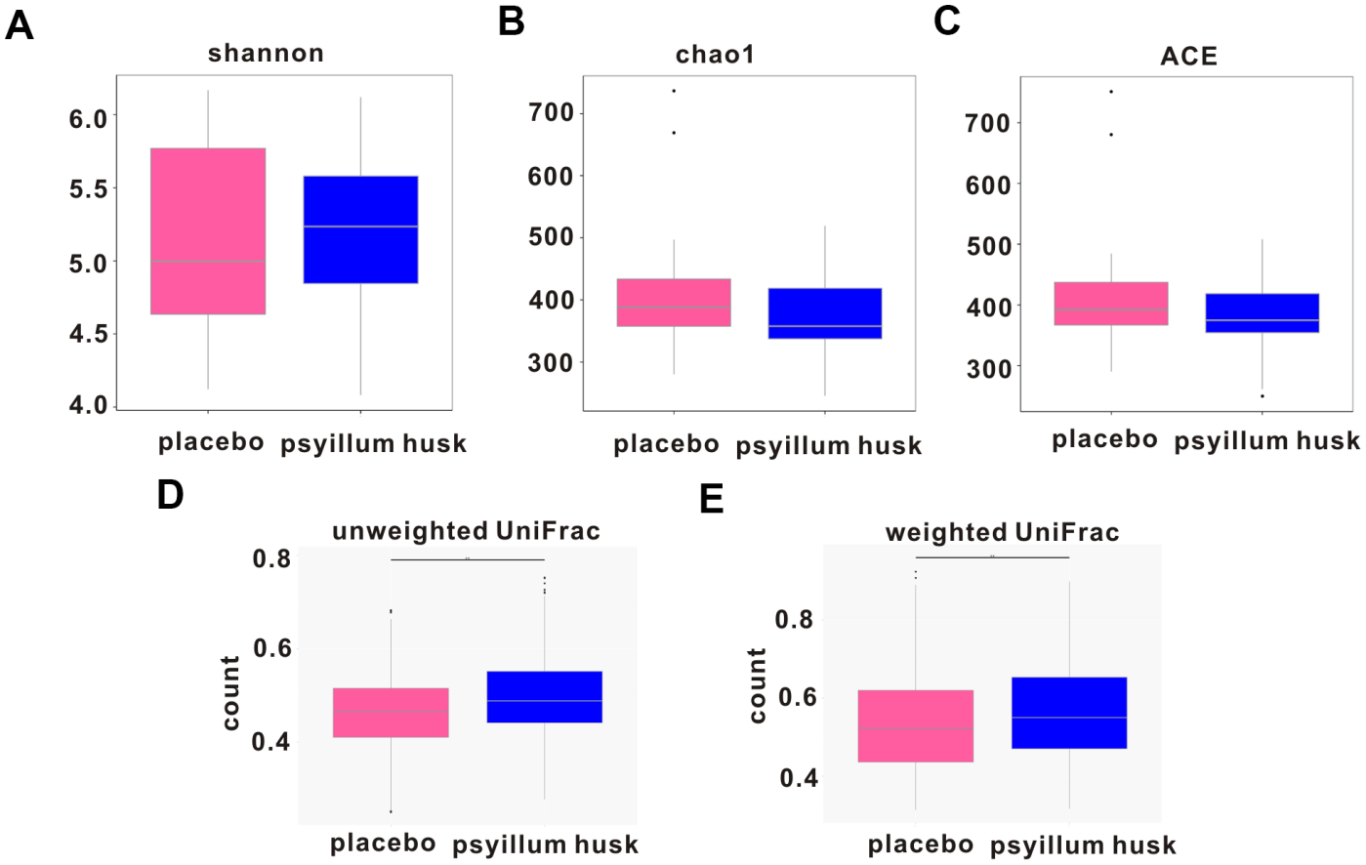
**Changes in intestinal microbe diversity during psyllium husk intervention.** α-diversity is indicated by the Shannon (**A**), Chao (**B**), and ACE diversity (**C**) indices, and β-diversity is indicated by unweighted (**D**) and weighted (**E**) UniFrac between the two groups.

To further evaluate how changes in microbial community structure differed between the two groups, we measured microbial α-diversity using the Shannon, Chao, and ACE diversity indices. Results showed that the between-group difference was not statistically significant (*P* = 0.938, *P* = 0.269, and *P* = 0.469, respectively; Figure 2A–2C). By contrast, β-diversity analysis showed that total diversity was significantly different (*P* = 1.67e-05 and *P* = 0.005 for unweighted and weighted UniFrac, respectively; Figure 2D, 2E).

**Figure 3:**
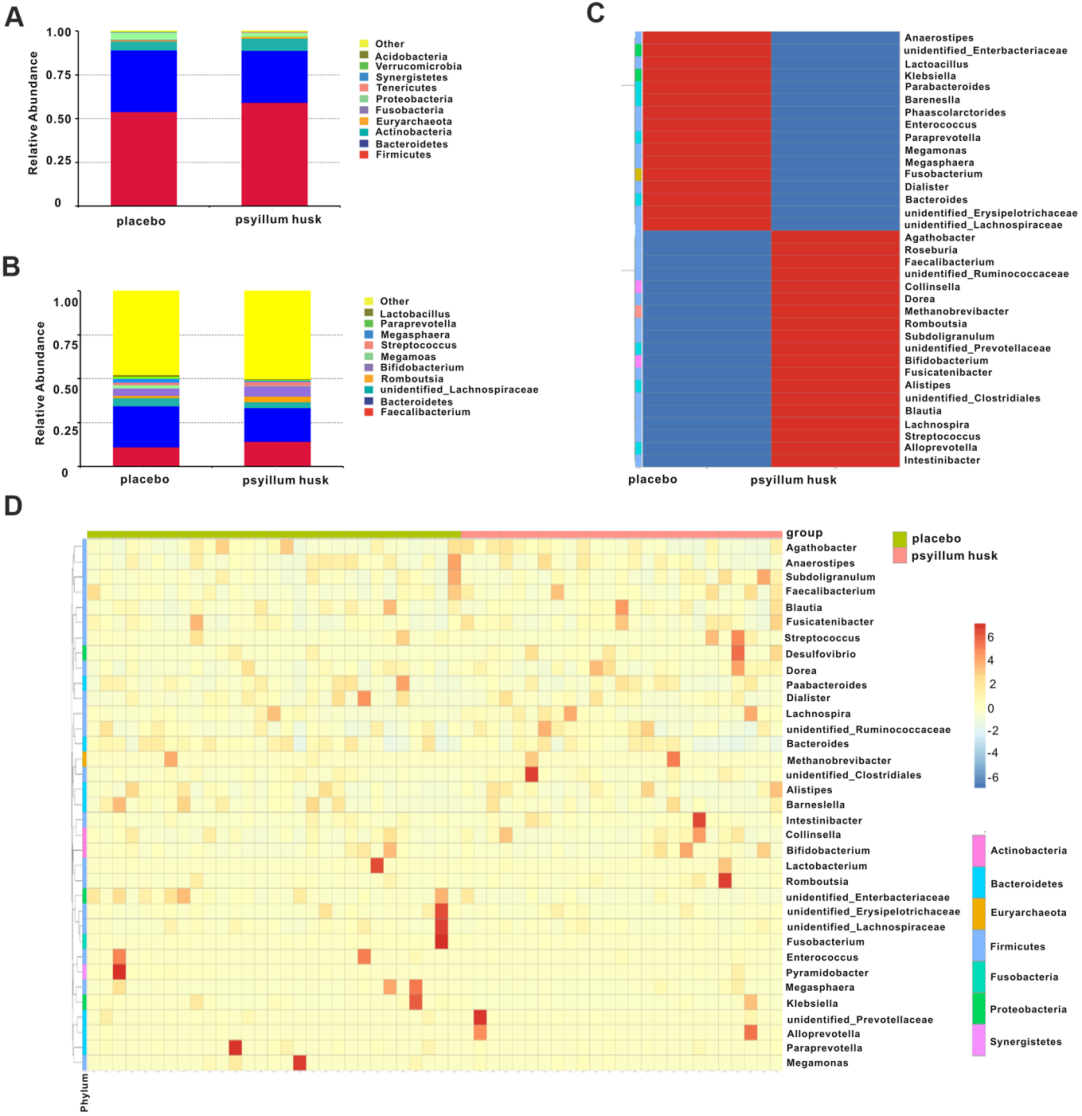
**Relative abundance of microbial communities at the phylum and genus levels in both groups.** (**A**) The average relative abundance of gut microbiota at the phylum level is shown by bar plot in each group. (**B**) Box plot showing relative abundances of microbial communities at the genus level in both groups. The cluster heatmap shows the differences in gut microbiota at the genus level between the groups (**C**) and among all samples (**D**).

To clarify the effect of psyllium on the intestinal flora of constipated women of reproductive age, we conducted a bar-plot analysis. A total of 18 different microbial phyla were detected in all samples; the top 10 gut microbes at the phylum level in both groups are shown in Figure 3A. *Firmicutes* was the most abundant phylum, accounting for 53.93% and 59.09% in the placebo and psyllium husk groups, respectively. *Bacteroidetes* constituted the second most common phylum present in the placebo and psyllium husk groups (35.14% vs. 25.78%, respectively). *Actinobacteria* was the third most common, accounting for 5.14% and 7.04% in the placebo and psyllium husk groups, respectively. *Fusobacteria* (0.55% vs. 0.02%), *Proteobacteria* (4.00% vs. 2.29%), *Synergistetes* (0.14% vs. 0.03%), *Verrucomicrobia* (0.19% vs. 0.09%), and *Acidobacteria* (0.04% vs. 0.00%) were more abundant in the placebo group than in the psyllium husk group. Meanwhile, *Euryarchaeota* (0.51% vs. 1.01%) and *Tenericutes* (0.30% vs. 0.60%; Figure 3A) were increased in the psyllium husk group. These results indicated that overall microbial composition at the phylum level differed significantly between the two groups.

At the genus level, we detected a total of 261 bacterial taxa, and we found that microbial composition differed significantly between the two groups. Per our analysis of the relative abundance of bacterial genera > 0.1%, the top 10 abundances are shown in Figure 3B, as follows for the placebo and psyllium husk group, respectively: *Faecalibacterium* (10.96% vs. 14.19%), *Bacteroides* (23.40% vs. 19.22%), *unidentified_Lachnospiraceae* (4.79% vs 3.35%), *Romboutsia* (1.21% vs. 3.20%), *Bifidobacterium* (4.19% vs. 5.78%), *Megamonas* (1.90% vs. 0.34%), *Streptococcus* (1.55% vs. 2.33%), *Megasphaera* (2.13% vs. 0.65%), *Paraprevotella* (1.19% vs. 0.31%), and *Lactobacillus* (0.86% vs. 0.54%). Our genus level analysis revealed that some strains were significantly increased in the control group. We used species-abundance cluster heatmap analysis to visualize the corresponding abundance relationship between samples and bacterial communities at the genus level (Figure 3C, 3D), which confirmed the results of our bar-plot analysis.

**Figure 4:**
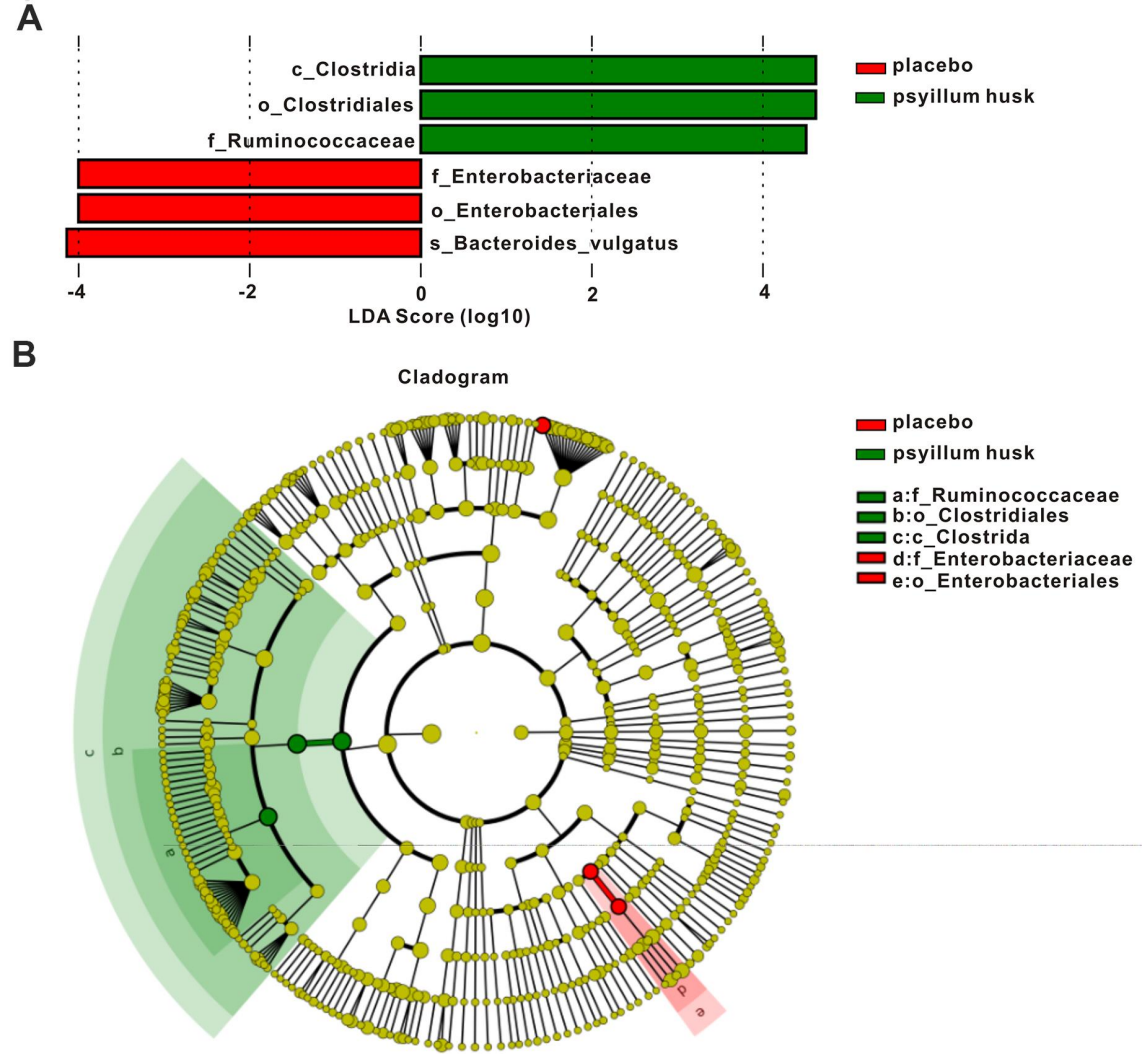
**LEfSe analysis of gut microbiotal bacteria.** (**A**) LDA score histogram of differentially abundant genera (LDA > 4, *P* < 0.05). (**B**) Cladograms display different species between the two groups. Species without significant differences are uniformly colored in yellow. Red nodes indicate the microbial groups that played important roles in the placebo group; green nodes indicate those that played important roles in the psyllium husk group. The diameter of each circle is proportional to its relative abundance.

To further determine whether specific individual bacterial taxa were differentially enriched between the placebo and psyllium husk groups, we applied LEfSe analysis, which couples LDA with effect-size measurements. As shown in Figure 4A, the following six taxa showed differentiated distributions with LDA scores >4: *f_Enterobacteriaceae*, *o_Enterobacteriales,* and *s_Bacteroides_vulgatus* were significantly more abundant in fecal samples from the placebo group, while we identified significant enrichment in *c_Clostridia*, *o_Clostridiales,* and *f_Ruminococcaceae* in the psyllium husk group.

Differential enrichment of specific bacteria is shown via cladograms based on an LDA = 4 for pairwise comparisons. According to this analysis, there was one family, one class, and one order in the psyllium husk group; and as well as one family and one order in the placebo group, that were differentially abundant between the two groups (Figure 4B). These results indicated that psyllium husk may be a significant factor in shaping gut microbiotal composition, possibly relieving the symptoms of constipation by reshaping intestinal flora composition in constipated women of reproductive age.

### Correlation network analysis and random forest analysis of microbiota abundance

**Figure 5:**
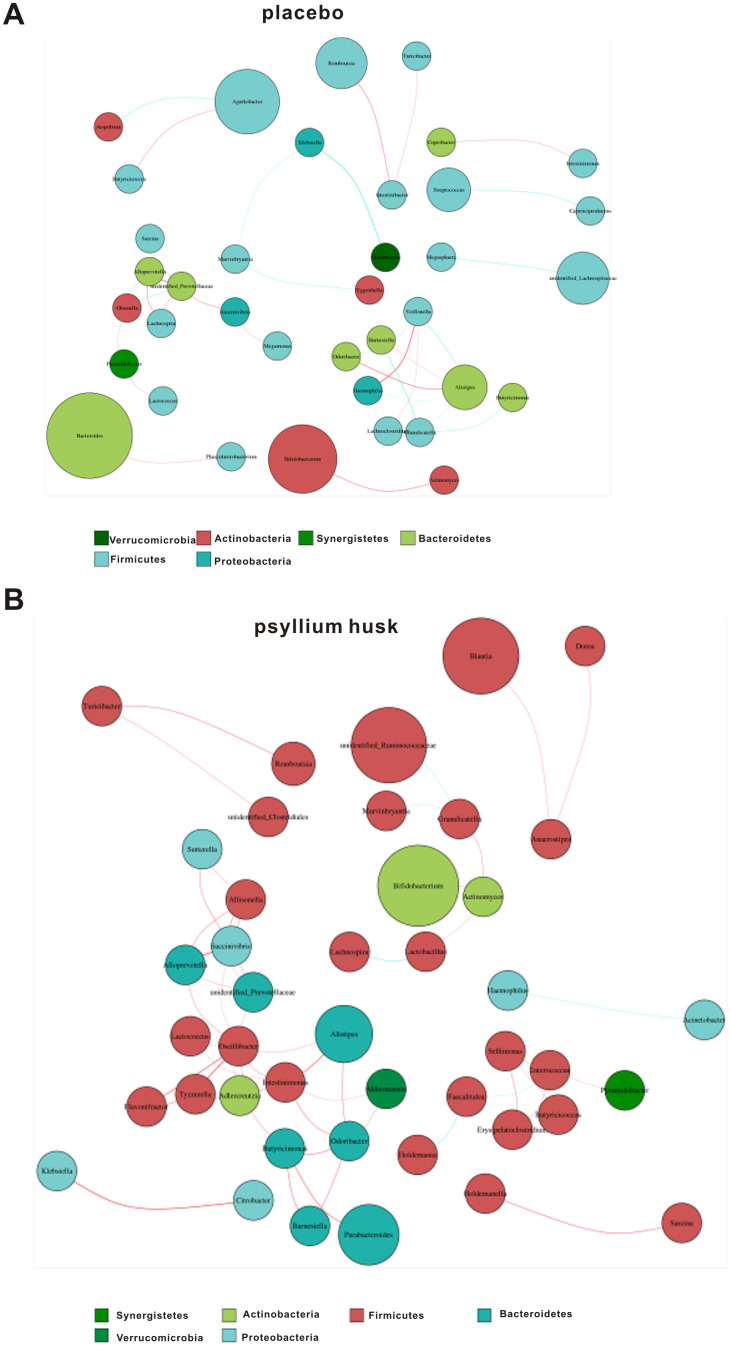
Correlation network analysis in the placebo (**A**) and psyllium husk (**B**) groups. Different nodes represent different genera, and the node size represents the average relative abundance of each genus. Thickness of the connection between each nodes is positively correlated with the absolute value of the correlation coefficient of species interaction (red: positive correlation; blue: negative correlation).

We further performed correlation network analysis to evaluate whether psyllium husk was related to changes in the correlation and constructive interaction structures of gut microbiota. The network results showed that samples from the placebo group had a smaller network diameter (ND; 6 vs. 10), lower graph density (GD; 0.014 vs. 0.006), and lower average degree (AD; 1.056 vs. 2.545) than those from the psyllium husk group (Figure 5). This indicated greater significant correlations and clustering of OTUs samples in the psyllium husk group.

**Figure 6:**
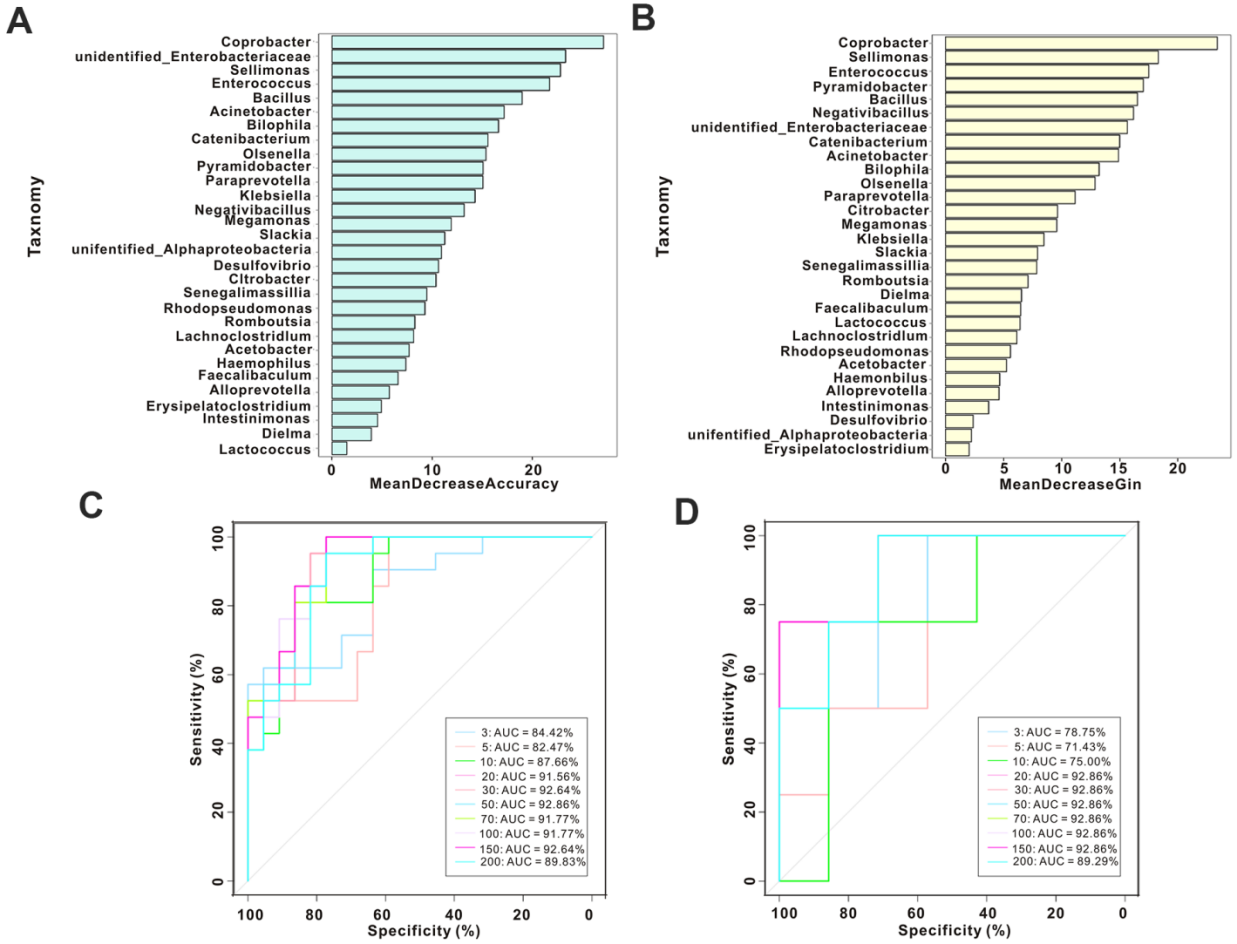
**Gut microbiotal signatures distinguish the placebo group from the psyllium husk group.** (**A**, **B**) We screened out important species using MeanDecreaseAccuracy and MeanDecreaseGin. (**C**) ROC analysis in the discovery cohort and with different numbers of bacteria. (**D**) ROC analysis in the validation cohort and with different numbers of bacteria.

Next, we performed random forest analysis using the relative abundances of the microbial populations to predict the potential of bacterial taxa in constipated women of reproductive age. Based on the species abundance at genus levels shown in this analysis, we selected different numbers of species by gradients to construct a random forest model. We screened out important species using MeanDecreaseAccuracy and MeanDecreaseGin (Figure 6A, 6B), cross-validated each model (10-fold), and constructed corresponding ROC curves. *Coprobacter* and *Sellimonas* in combination with *Enterococcus* achieved an area under the curve (AUC) value of 84.42% in the discovery model; *Coprobacter* and *Bacillus* in combination with *Sellimonas* achieved an AUC value of 78.57% in the invalidation model (Figure 6C, 6D).

### Predictive microbial functional profiling

**Figure 7:**
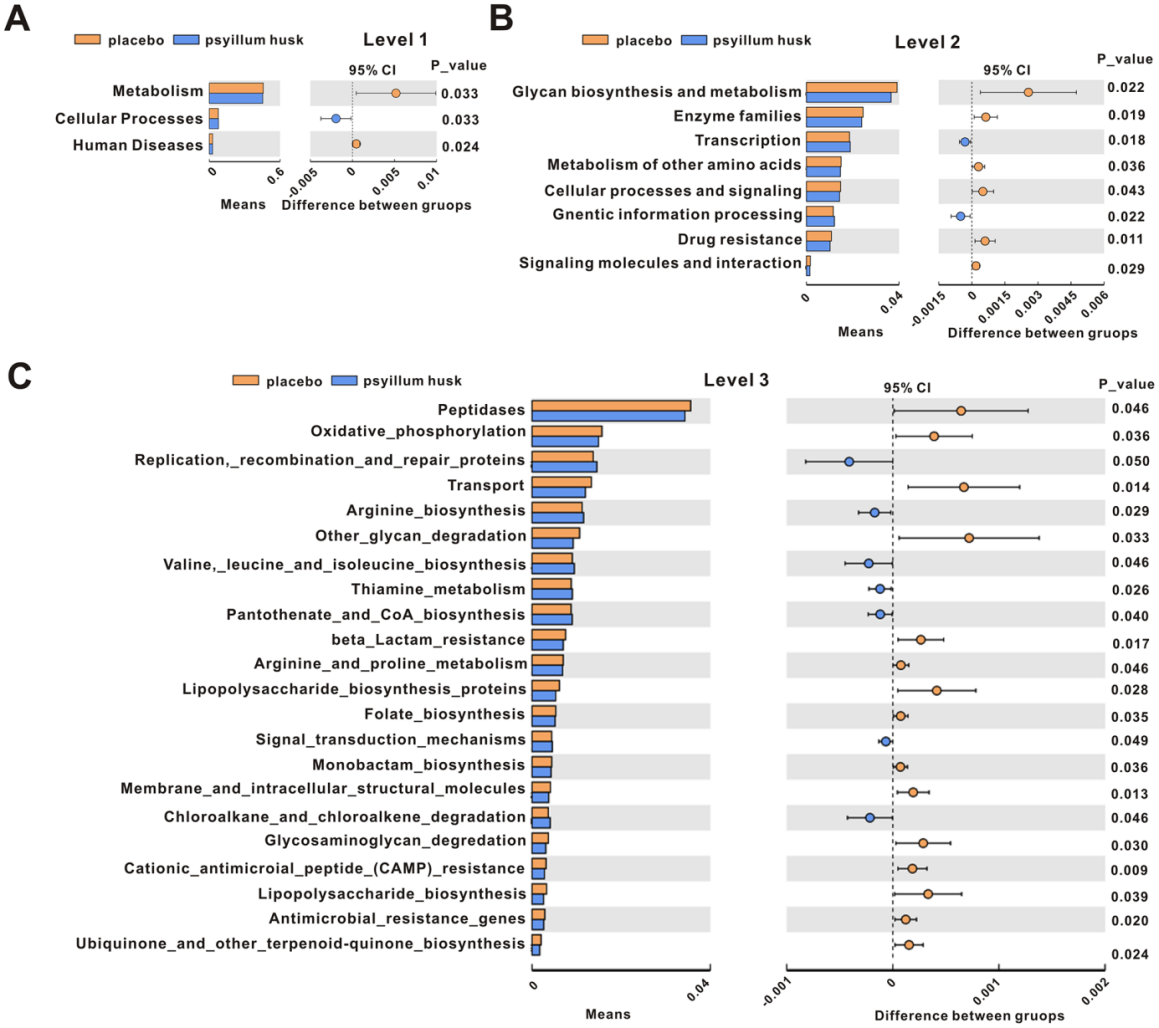
KEGG signaling pathways were compared between the placebo and psyllium husk groups at levels 1 (**A**), 2 (**B**), and 3 (**C**), as analyzed by Student’s *t* test (*P* < 0.05). CI: confidence interval.

Tax4fun (http://tax4fun.gobics.de/) is a software package that can predict the functions of microbial communities based on 16S rRNA datasets [20]. It has been shown to provide a good approximation of functional profiles for data obtained from the metagenome. Therefore, in our present study, we used Tax4Fun analysis to reveal differential functional profiles of communities between the placebo and psyllium husk groups. At level 1, the proportion of metabolic and human disease-related sequences was significantly decreased (*P* < 0.05) in the fecal microbiome of the psyllium husk group, while cellular processes were significantly increased (Figure 7A). At level 2, the functional categories associated with glycan biosynthesis and metabolism, enzyme families, metabolism of other amino acids, cellular processes and signaling, drug resistance, and signaling molecules and interaction were enriched in the placebo group, while transcription and genetic information processing were increased in the psyllium husk group (Figure 7B). At level 3, we found that 15 pathways (peptidases, oxidative_phosphorylation, transport, other_glycan_degradation, bata-lactam_resistance, arginine_and_proline_metabolism, lipopolysaccharide_biosynthesis_proteins, folate_biosynthesis, monobacyam_biosynthesis, membrane_and_intracellular_structural_molecules, glycosaminoglycan_degradation, cationic_antimicrobial_peptide_(CAMP)_resistance, lipopolysaccharide_biosynthesis, antimicrobial_resistance_genes, and ubiquinone_and_other_terpenoid-quinone_biosynthesis) were significantly increased in the placebo group, while 7 pathways (replication,_recombination_and_repair_proteins, arginine_biosynthesis, valine,_leucine_and_isoleucine_biosynthesis, thiamine_metabolism, pantothenate_and_CoA_biosynthesis, signal_transduction_mechanisms, and chloroalkane_and_chloroalkene_degradation) were significantly enriched in the psyllium husk group (Figure 7C).

According to the functional annotations and abundance information of samples in the SILVA database, we selected the 35 most abundant features and their abundance information in each sample to draw a heatmap and clustered the functional differences. At level 1 (Supplementary Figure 1A), the functional categories associated with organismal_systems, human_disease, and metabolism were enriched in the placebo group, while genetic_information_processing, environmental_information_processing, and cellular_processes were increased in the psyllium husk group. At level 2 (Supplementary Figure 1B), we found that 21 pathways in the placebo group and 14 pathways in the psyllium husk group were significantly increased. At level 3 (Supplementary Figure 1C), we found significant increases of 23 pathways in the placebo group and 12 pathways in the psyllium husk group.

## DISCUSSION

Previous studies have shown that psyllium husk relieves constipation symptoms by improving fecal water content, transit time, and volume/frequency of BMs, as well as the concentration of short chain fatty acids (SCFAs) [18, 21]. Our present study represents the first to investigate constipation symptoms and patterns of gut microbiotal composition and function in a psyllium husk intervention model in constipated women of reproductive age using 16S rRNA gene sequencing.

Our present study revealed the clinical significance of improvements in symptom of constipation in such women after four weeks of psyllium intervention. These results are consistent with improvements of symptoms in adults with constipation, children with constipation, or constipation in patients with type II diabetes [18, 22, 23]. Psyllium fibers improve clinical symptoms of constipation mainly by increasing water retention capability throughout intestinal transit, facilitating a water-rich colonic environment, and increasing the softness and volume of feces [24, 25]. Importantly, intestinal microbes play an important role in the beneficial effects of psyllium husk; however, the underlying mechanisms of this association have remained unclear.

Therefore, in our present study, we first investigated microbial α- and β-diversity to determine between-group differences in microbial community structure. Changes in the psyllium husk group microbiota mainly featured increases in *Faecalibacterium*, *Romboutsia*, *Streptococcus*, and *Bifidobacterium* abundances and decreases in *Bacteroides*, *unidentified_Lachnospiraceae*, *Megamonas*, *Megasphaer*, *Paraprevotella*, and *Lactobacillus* abundances. *Faecalibacterium* is a butyrate-producing bacterium associated with fiber intake; its abundance was increased after psyllium intervention in our present study, which is consistent with previous studies [26]. Fateh et al. have shown that synbiotics containing *Streptococcus* help improve the symptoms of male patients with functional constipation [27]. A synergic mixture of *Bifidobacterium species* and other species has been confirmed to be vital for clinical characteristics of functional constipation patients [27]. In our present study, the abundance of *Bifidobacterium* was increased in the psyllium husk group. Jalan et al. showed that *Lachnospiraceae* abundance was decreased in constipated patients compared with that of healthy control group (0.13% vs. 0.58, respectively) but was increased after psyllium husk intervention (baseline vs. psyllium: 0.11% vs. 0.20%) [18]. However, our study found that the relative richness of *Lachnospiraceae* was decreased in the psyllium husk group. This inconsistency in results may have been due to differences in sex and age across studies. In addition, most studies have shown that *Lactobacillus* is lower in patients with chronic constipation, and randomized controlled trial (RCT) results indicate that probiotics containing *Lactobacillus* relieve gastrointestinal symptoms in chronic constipation [28–30]. In our present study, patients with constipation had high levels of *Lactobacillus*, which were decreased after with psyllium husk intervention; this result may have been due to our being all women.

To determine whether intestinal flora structure was also changed in the psyllium husk group, we conducted correlation network analysis and found further significant correlations and more clustering of OTUs compared with samples from the placebo group. These results indicated that altered correlation network structure following psyllium husk intervention might be involved in the different compositions and metabolic functions of the gut microbiota.

Tax4Fun analysis was used to reveal differences in functional profiles of communities between the placebo and psyllium husk groups in our present study. KEGG annotations in the psyllium husk group were mainly involved in metabolism, including glycan biosynthesis and metabolism, arginine biosynthesis and metabolism, thiamine metabolism, proline metabolism, and metabolism of other amino acids. This observation is consistent with the consensus that constipation is highly correlated with metabolic disorders [31, 32]. These results indicated that changes in gut microbiotal function might contribute to the pathogenesis of constipation via the metabolic signaling pathways. Previous studies have also shown that psyllium can improve the metabolism of patients with constipation mainly by reducing cholesterol levels and increasing high-density lipoprotein cholesterol (HDLC) levels [23].

There were still some limitations in this study. First, the focus of this study was on constipated female patients of childbearing age. The age of patients should be further narrowed and limited to the optimal reproductive age in future studies. Second, we used the fecal microbiome to indicate changes in the intestinal microbiome and focused only on microbiotal composition and function; therefore, metabolomic and transcriptomic data should be further explored. Moreover, *in vitro* studies are needed to verify that psyllium husk affects specific metabolic signaling pathways by altering the gut microbiota. Therefore, it is necessary for further research to verify the effects reported in our present study.

Taken together, our findings provide new insights into the composition and function of intestinal microbiota in constipated women of reproductive age. More importantly, disturbances in these gut microbiota were found to be related to metabolism, indicating that the symptoms of constipation can be alleviated by changing the metabolism of intestinal microflora. Further studies are needed to elucidate the causal relationships between intestinal microbiota and constipation in women of childbearing age.

## MATERIALS AND METHODS

This study was approved by the Ethics Committee of Shanxi Bethune Hospital (Taiyuan, China; Certificate No. XYLL-2019-124), and all participants gave informed consent.

### Patients and trial design

This was a single-blinded, randomized, placebo-controlled trial of 54 women of reproductive age with symptoms of chronic constipation (meeting Rome IV diagnosis standards). Most of the patients were recruited from Shanxi Bethune Hospital. Patients were included if they satisfied the following conditions: (a) had a BM frequency of <3 times/week over the preceding 3 months; (b) were women of reproductive age (18–49 years); and (c) had a BMI within a normal range from 18.5-24 kg/m^2^ [33]. Patients were excluded based on any of the following: (a) were children, men, pregnant, or postmenopausal; (b) had metabolic or neuropsychiatric conditions, cancer, diabetes, Alzheimer disease, or Parkinson disease; (c) had history of intestinal surgeries; or (d) were taking antibiotics, probiotics, prebiotics, nonsteroidal anti-inflammatory drugs (NSAIDs), opioids, traditional Chinese medicine (TCM), proton pump inhibitors (PPIs), or histamine receptor antagonists during the preceding month before sample collection. After participants provided written informed consent, they were advised at the first visit to maintain their daily lifestyles, diets, and physical activities throughout the study. They were asked to take a placebo or psyllium husk (Supplementary Table 1) with a cup of warm water twice a day (30 min before breakfast and dinner). The placebo was consistent with psyllium husk except that it did not contain the seed shell of *Plantago rotundus*. Participants used a stool diary to record the quantity and characteristics of their stools and to subjectively evaluate the effectiveness of the interventional agent. At the end of week 1, we contacted them to assess compliance with the intervention. Clinicians evaluated the possible side effects of the intervention, as well as efficacy parameters, both at baseline and weekly after the start of treatment. The entire experiment lasted 4 weeks. The 60 participants were randomly assigned to one of two treatment groups. By the end of the trial, three patients had been lost to follow-up; two in the psyllium husk group had withdrawn from the study, while one in the placebo group had withdrawn from the study (Supplementary Figure 2). Please refer to the Supplemental Data table for more clinical characteristic information about the participants.

### Randomization scheme

A total of 60 participants who met the research criteria were recruited and randomly assigned to two treatment groups. Participants were randomly assigned to a preexisting list generated according to a computer program, and the assignment of each group was concealed in an opaque sealed envelope and kept by a dedicated person.

### Sample collection

Subjects’ fecal samples were collected into tubes pre-filled with Stool DNA Stabilizer (PSP^R^ Spin Stool DNA Plus Kit; Invitek Molecular, Berlin, Germany). Within 3 days, we transferred the tubes to −80° C storage until the next processing step.

### 16S ribosomal ribonucleic acid (rRNA) gene sequencing analysis

We performed 16S rRNA sequencing analysis of human feces as previously described [34, 35]. Briefly, we extracted fecal microbial DNA from 54 samples (29 samples from the placebo group and 25 samples from the psyllium husk group) using an E.Z.N.A. Stool DNA Kit (Omega Bio-tek, Inc., Norcross, GA, USA) per the manufacturer’s protocols. Subsequently, we conducted polymerase chain reaction (PCR) amplification of bacterial V3–V4 hypervariable regions of the 16S rRNA gene using the primers PF 5′-CCTAYGGGRBGCASCAG-3′ and PR 5′-GGACTACNNGGGTATCTAAT-3′. The barcode sequences and FLX Titanium adaptors were incorporated into the primers. Then, we pooled the purified amplification products in equal molar amounts and sequenced the paired ends on an Illumina MiSeq platform (Illumina, Inc., San Diego, CA, USA). Raw data were demultiplexed, quality filtered, and spliced to obtain clean data. Effective sequences were clustered into OTUs with a 97% identity cutoff, and chimeric sequences were removed via the UCHIME algorithm (https://www.drive5.com/usearch/manual/uchime_algo.html). We classified OTUs using the Ribosomal Database Project (RDP) classifier algorithm (http://rdp.cme.msu.edu/) and the SILVA database (https://www.arb-silva.de/).

### Bioinformatic analysis

We analyzed 16S rRNA gene diversity using the Quantitative Insights Into Microbial Ecology (QIIME) tool. To determine α-diversity, we used the Chao, Shannon, and Abundance-based Coverage Estimator (ACE) diversity indices [36]; both unweighted and weighted UniFrac phylogenetic distance matrices were used to evaluate β-diversity [37, 38]. Linear discriminant analysis (LDA) of effect size (LEfSe), Student’s *t* test, and the Wilcoxon rank-sum test were used to analyze differences in bacterial abundances at different bacterial classification levels. Relationships among microbial communities were visualized by a network [39]. Random forest is a classic machine learning model based on a classification tree algorithm, which we performed as previously described [40]. Briefly, based on the analysis of species richness, we constructed a random forest model by selecting different numbers of species by gradients at different classification levels. The important species were selected by MeanDecreaseAccuracy and MeanDecreaseGin. Then, we cross validated each model and drew ROC curves.

### Statistical analyses

For characteristics of constipated patients, data are described as the mean ± standard deviation (SD) or as the median (percentage). We used Wilcoxon rank-sum test, independent sample *t* test, Pearson’s *χ*^2^ test, or Fisher’s exact test to evaluate differences between the groups. *P* < 0.05 was considered statistically significant. We performed all statistical analyses using SPSS version 19.0 (IBM Corp., Armonk, New York, USA).

## Supplementary Material

Supplementary Figures

Supplementary Tables
