# Correction for: The effects of psyllium husk on gut microbiota composition and function in chronically constipated women of reproductive age using 16S rRNA gene sequencing analysis

**DOI:** 10.18632/aging.203991

**Published:** 2022-03-30

**Authors:** Chuanli Yang, Shuai Liu, Hongxia Li, Xinshu Bai, Shuhua Shan, Peng Gao, Xiushan Dong

**Affiliations:** 1 Department of General Surgery, Shanxi Bethune Hospital, Shanxi Academy of Medical Sciences, Taiyuan, China; 2 Graduate School, Shanxi Medical University, Taiyuan, China; 3 Shanxi Provincial Cancer Hospital, Taiyuan, China; 4 Key Laboratory of Chemical Biology and Molecular Engineering of National Ministry of Education, Institute of Biotechnology, Shanxi University, Taiyuan, China; 5 BGI Life Science Research Institution, Shenzhen, China

**Keywords:** women of reproductive age, chronic constipation, gut microbiota, 16S rRNA gene sequencing, metabolism

Original article: Aging. 2021; 13:15366–15383. https://doi.org/10.18632/aging.203095

**This article has been corrected:** The authors corrected the sentence in the **Introduction** referring to women of childbearing age, where it was wrongly written as “…between the ages of 15 and 49 years.” It should have read  “…between the ages of 18 and 49 years.” This correction reflects the age of the actual patients, does not change the content of the publication and does not affect the conclusion of this research.

New text of the corrected paragraph is presented below.

## Introduction

Chronic constipation is one of the most common symptoms worldwide; it can occur by itself or can be secondary to other medical conditions [1]. The main characteristics of chronic constipation are difficult passage of stool, reduced frequency of bowel movements (BMs), and a feeling of incomplete defecation. Most previous studies have shown that the prevalence of constipation is 12–19% in adults living in North America [2, 3], whereas this prevalence is 26% in women and 16% in men over the age of 65 [4, 5]. In addition, among people aged ≥84 years, the prevalence of constipation is 34% in women and 26% in men [6–8]. Especially during late pregnancy, the risk of constipation is higher due to reduced intestinal movement, significant hormonal changes, and delayed bowel emptying [9, 10]. However, most of the current research on constipation has focused on all adults, and has only rarely focused specifically on women of reproductive age (women of childbearing ability between the ages of 18 and 49 years) alone. Therefore, there is an urgent need to explore the impact of constipation in such women.

