# Supplementary material for: The effects of psyllium husk on gut microbiota composition and function in chronically constipated women of reproductive age using 16S rRNA gene sequencing analysis
Source: Aging (Albany NY). 2021 Jun 3;13(11):15366–83. doi: 10.18632/aging.203095 (PMC8221300; doi:10.18632/aging.203095)
Supplement: Supplementary Figures [file aging-13-203095-s001.pdf]

SUPPLEMENTARY FIGURES

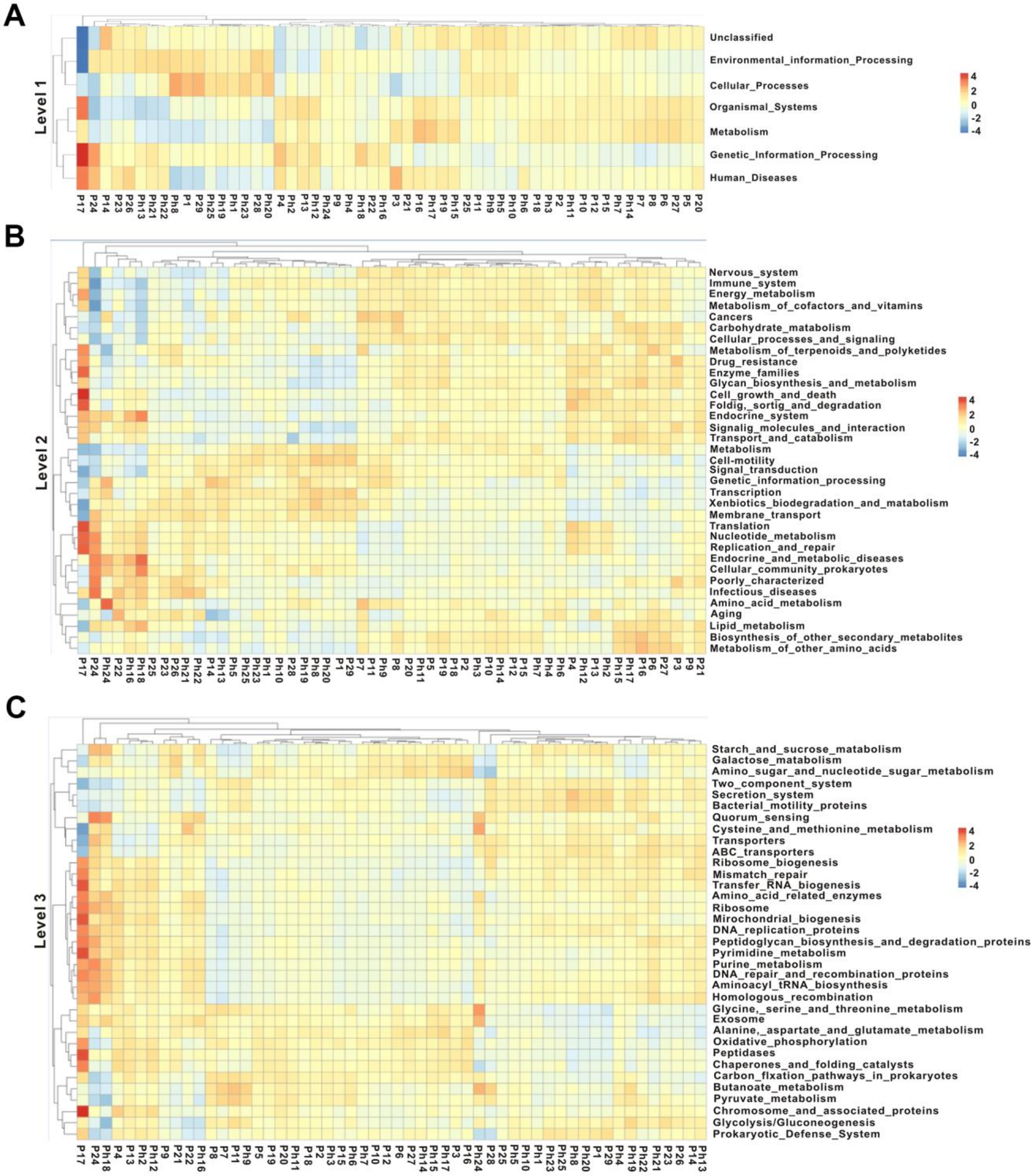

Supplementary Figure 1. Cluster heatmap showing signaling pathways of all samples at levels 1 (**A**), 2 (**B**), and 3 (**C**).

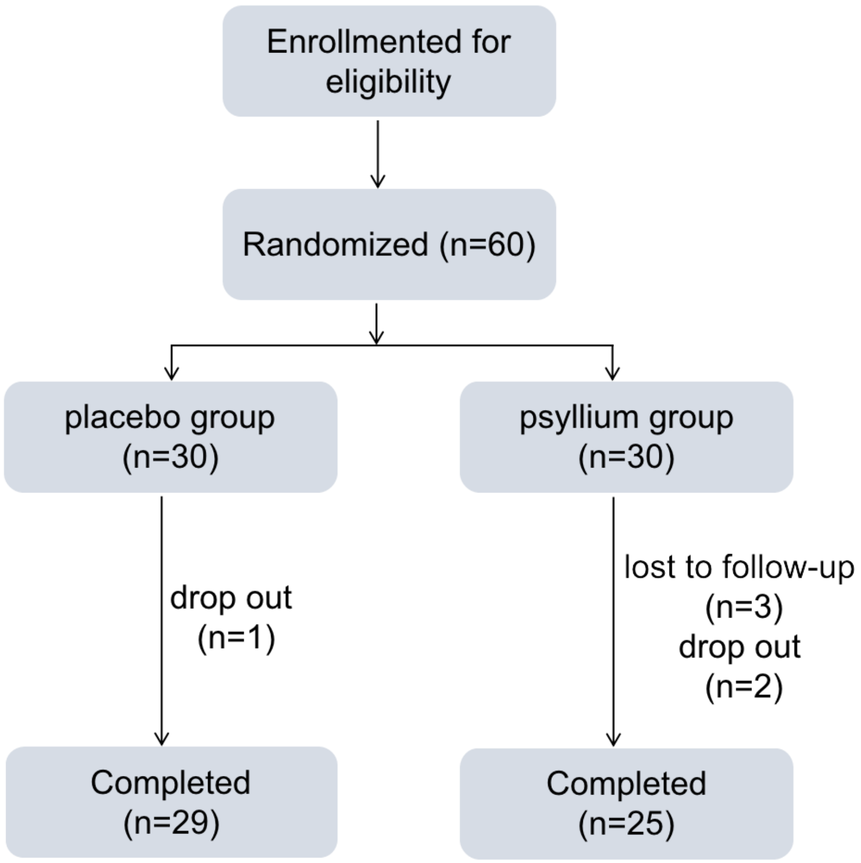

Supplementary Figure 2. Experimental design for the study.
