# Supplementary material for: The effects of psyllium husk on gut microbiota composition and function in chronically constipated women of reproductive age using 16S rRNA gene sequencing analysis
Source: Aging (Albany NY). 2021 Jun 3;13(11):15366–83. doi: 10.18632/aging.203095 (PMC8221300; doi:10.18632/aging.203095)
Supplement: Supplementary Tables [file aging-13-203095-s002.pdf]

SUPPLEMENTARY TABLES

Supplementary Table 1. Nutrient composition of psyllium husk.

Characteristics	Per 100 g	NRV %
Energy	883 KJ	11 %
Protein	0 g	0 %
Fat	0 g	0 %
Carbohydrate	1536 g	5 %
Fiber	77.2 g	309 %
sodium	38 mg	2 %

Supplementary Table 2. Characteristics of constipated patients of reproductive age by treatment group at baseline.

Characteristics	placebo (x ± SD)	psyllium husk (x ± SD)	*P*-Value
ALT	15.377 (6.155)	14.573 (6.939)	0.686
AST	21.000 (7.399)	20.443 (7.875)	0.936
TBIL	12.070 (3.776)	11.170 (4.284)	0.393
DBIL	2.653 (0.748)	2.260 (0.866)	0.595
IBIL	9.417 (3.095)	8.910 (3.484)	0.364
ALB	44.239 (2.049)	45.000 (6.497)	0.104
TBA	2.700 (1.634)	2.717 (1.405)	0.599
CHO	3.969 (0.698)	4.244 (0.763)	0.537
TG	0.935 (0.470)	1.148 (1.039)	0.156
HLD	1.430 (0.324)	1.413 (0.271)	0.509
LDL	2.220 (0.466)	2.409 (0.527)	0.342
